# Meteorological Report for February

**Published:** 1880-03

**Authors:** H. Hall

**Affiliations:** Corp’l. Signal Service U.S. A.; Atlanta, Ga.


### METEOROLOGICAL REPORT FOR FEBRUARY, 1880.

| RAIN. | | Mean daily Barometer. | Mean daily Thermometer | Mean daily Humidity. | Maximum Thermometer | Minimum Thermometer | Prevailing direction of Wind. | Maximum hourly velocity of Wind, (Daily.) |
|---|---|---|---|---|---|---|---|---|
| DATE | IN. | | | | | | | |
| 1 | 0 | 30.107 | 44.5 | 43.3 | 55 | 39 | N. W. | ......... |
| 2 | 2.10 | 29 724 | 34 2 | 89.7 | 44 | 32 | E. | ......... |
| 3 | 55 | 29.926 | 36.0 | 66.3 | 45 | 31 | W. | ......... |
| 4 | 0 | 30 216 | 37.0 | 53.0 | 47 | 31 | W. | ......... |
| 5 | 0 | 30.349 | 39.7 | 45.7 | 48 | 32 | N. W. | ......... |
| 6 | 0 | 30.396 | 43 7 | 43.0 | 57 | 35 | N. W. | ......... |
| 7 | 0 | 30.471 | 46 2 | 41.0 | 55 | 35 | N. W. | ......... |
| 8 | 0 | 30.387 | 45.0 | 44.3 | 53 | 39 | E. | ......... |
| 9 | 0 | 30.261 | 53 0 | 40.3 | 61 | 38 | N. | ......... |
| 10 | 0 | 30 221 | 58.0 | 56.7 | 66 | 49 | S. E. | ......... |
| 11 | 0 | 30.147 | 58.7 | 65.0 | 65 | 51 | S. E. | ......... |
| 12 | 08 | 29.904 | 63.7 | 71.7 | 70 | 56 | S. E. | ......... |
| 13 | 25 | 29.753 | 59.0 | 73.3 | 69 | 52 | W. | ......... |
| 14 | 0 | 29.966 | 49.5 | 61.0 | 57 | 42 | N. W. | ......... |
| 15 | 0 | 30 234 | 44.0 | 49.3 | 51 | 35 | N. W. | ......... |
| 16 | 0 | 30 447 | 47 | 39.3 | 58 | 35 | E | ......... |
| 17 | 0 | 30 357 | 59.0 | 60 6 | 65 | 45 | S E. | ......... |
| 18 | 13 | 30 203 | 58.3 | 83.6 | 64 | 55 | S. | ......... |
| 19 | 0 | 30.351 | 45.5 | 45.6 | 55 | 40 | N. W. | ......... |
| 20 | 0 | 30 348 | 46.0 | 41.3 | 51 | 36 | E. | ......... |
| 21 | 30 | 29.761 | 46 5 | 74.6 | 54 | 42 | N. W. | ......... |
| 22 | 0 | 29.963 | 53.5 | 53.0 | 63 | 38 | S. | ......... |
| 23 | 0 | 30 076 | 55.7 | 40.0 | 63 | 51 | W. | ......... |
| 24 | 0 | 30.108 | 55.7 | 31.7 | 65 | 44 | S. E. | ......... |
| 25 | 0 | 29.958 | 51.7 | 57.3 | 70 | 46 | S. | ......... |
| 26 | 11 | 29.974 | 63.2 | 78.0 | 67 | 60 | S. W. | ......... |
| 27 | 0 | 30 088 | 62.5 | 83.3 | 71 | 60 | S. W. | ......... |
| 28 | 0 | 29.971 | 66.2 | 66.0 | 74 | 59 | S. W. | ......... |
| 29 | 09 | 30.048 | 56.0 | 85.0 | 67 | 51 | S. W. | ......... |
| 30 | 0 | ......... | ...... | ...... | ... | ... | ......... | ......... |
| 31 | 0 | ......... | ...... | ...... | ... | ... | ......... | ......... |
| Sums | 2.86 | 873.733 | 146.85 | 168.29 | 19.13 | 15.56 | ......... | ......... |

Highest Barometer, 30.518, on the 7th.
Lowest Barometer, 29.561, on the 2nd.
Monthly range of Barometer, 0.957.
Highest Temp. 74°, on 28th. Lowest Temp. 31°, on 3d and 4th.
Monthly range of Temperature, 43°.
Greatest daily range of Temperature, 25°, on the 22nd.
Least daily range of Temperature, 7°, on the 26th.
Mean of Maximum Temperatures, 50°4.
Mean of Minimum Temperatures, 43°7.
Mean daily range of Temperature, 15°7. Total rainfall, 3.11 inches.
Prevailing wind, N. W. Total movement of wind, 75.96 miles.
Maximum velocity of wind and direction, 36 miles per hour, E., 2nd.
Number of cloudy days on which no rain fell, 3.
Total number of days on which rain fell, 9.

H. HALL, Corp'l. Signal Service U. S. A.

Atlanta, Ga., Feb. 1, 1880.

